# Midfrontal Theta and Posterior Parietal Alpha Band Oscillations Support Conflict Resolution in a Masked Affective Priming Task

**DOI:** 10.3389/fnhum.2018.00175

**Published:** 2018-05-03

**Authors:** Jun Jiang, Kira Bailey, Xiao Xiao

**Affiliations:** ^1^Department of Basic Psychology, School of Psychology, Third Military Medical University, Chongqing, China; ^2^Department of Psychology, Ohio Wesleyan University, Delaware, OH, United States; ^3^School of Public Health and Management, Chongqing Medical University, Chongqing, China

**Keywords:** conflict, theta, alpha, facial expressions, fronto-parietal network, affective priming

## Abstract

Past attempts to characterize the neural mechanisms of affective priming have conceptualized it in terms of classic cognitive conflict, but have not examined the neural oscillatory mechanisms of subliminal affective priming. Using behavioral and electroencephalogram (EEG) time frequency (TF) analysis, the current study examines the oscillatory dynamics of unconsciously triggered conflict in an emotional facial expressions version of the masked affective priming task. The results demonstrate that the power dynamics of conflict are characterized by increased midfrontal theta activity and suppressed parieto-occipital alpha activity. Across-subject and within-trial correlation analyses further confirmed this pattern. Phase synchrony and Granger causality analyses (GCAs) revealed that the fronto-parietal network was involved in unconscious conflict detection and resolution. Our findings support a response conflict account of affective priming, and reveal the role of the fronto-parietal network in unconscious conflict control.

## Introduction

Numerous studies over the past several years have revealed that automatically evaluated affective information has a substantial impact on subsequent decision and judgment (Fazio et al., 1986; Murphy and Zajonc, 1993; Klauer and Musch, 2003; Dannlowski and Suslow, 2006; Frings and Wentura, 2008; Li et al., 2008; Bartholow et al., 2009; Gibbons, 2009; Jiang et al., 2013; Kiefer et al., 2017). This work has used various priming tasks, in which an affective prime precedes a positive or negative target stimulus and participants are asked to categorize the valence of the target as quickly and accurately as possible. The major finding from these priming tasks is an affective congruency effect; that is, the mean reaction times (RTs) and/or error rates (ERs) are higher in incongruent prime-target pairs compared to congruent ones. Importantly, the affective congruency effect is still present even though the primes are not consciously perceived by participants due to their brief duration and masking (e.g., Murphy and Zajonc, 1993; Greenwald et al., 1996; Dannlowski and Suslow, 2006; Li et al., 2008; Gibbons, 2009; Jiang et al., 2013; Kiefer et al., 2017). The occurrence of a subliminal affective congruency effect suggests that the unconsciously processed affective information can modulate ongoing evaluation and judgments even if this information is outside conscious awareness (for a review, see Klauer and Musch, 2003).

Semantic activation and response activation accounts have been proposed to explain the underlying mechanisms of the affective congruency effect (Klinger et al., 2000; Klauer and Musch, 2003; Bartholow et al., 2009; Eder et al., 2012; Goerlich et al., 2012; Kiefer et al., 2017). In the semantic activation account, affective priming is due to spreading activation, similar to semantic priming (e.g., Neely, 1991). The concept node associated with the active prime is activated in an associative and interconnected semantic network, resulting in pre-activation of the valence congruent targets via spreading activation, thus facilitating the processing of subsequent valence congruent targets (e.g., Spruyt et al., 2007). In contrast, the more recently proposed response activation account conceptualizes the affective congruency effect in terms of conflict, although the exact source(s) of this conflict remain uncertain (Bartholow et al., 2009; Goerlich et al., 2012). The response activation account holds that the affective prime could directly activate the corresponding response tendencies automatically based on the learned stimulus-response (S-R) mapping rule in particular if there is a fixed/small task sets, irrespective of the valence congruency of the prime and target (Klinger et al., 2000; Damian, 2001). Therefore, congruency between response tendencies for the prime and target facilitates responding to the target. Responding to incongruent targets would be slower due to conflict that occurs when the participants must overcome the prepotent response tendency to the prime in order to achieve the task goal of responding to the target. Previous studies provided mixed evidence for the above two accounts (see reviews in Eder et al., 2012; Kiefer et al., 2017). In the current study, we will mainly focus on the conflict account of affective priming.

The idea of conflict in affective priming has been confirmed by some electrophysiological studies using behavioral and event-related potentials (ERPs) measures. Using visible affective primes and targets, previous studies showed that the amplitude of the conflict monitoring related fronto-central N2 component and the response activation related lateralized readiness potential (LRP) were modulated by response conflict (Bartholow et al., 2009; Eder et al., 2012). Further, the semantic conflict related N400 component (Chen et al., 2010; Goerlich et al., 2012) was modulated by semantic conflict in affective priming tasks (Zhang et al., 2010; Eder et al., 2012; Kiefer et al., 2017).

While the previous studies have illustrated the important role of conflict during affective priming triggered by visible/conscious primes, whether conflict is triggered by invisible/unconscious primes has not been well established. More importantly, the neural oscillatory dynamics of conflict control during subliminal affective priming remains poorly understood due to lack of related studies. Nevertheless, studies on unconscious conflict with a masked priming paradigm can give us some insights. Using a Stroop/flanker-like masked priming task and non-affective stimuli, previous scalp electroencephalogram (EEG) studies showed that conflict could be induced by unconscious primes and this unconscious conflict results in a medial frontal theta band (usually 4–8 Hz) power increase and parieto-occipital alpha band (usually 8–12 Hz) power decrease (Jiang et al., 2015a,b). Further, the medial frontal theta and parieto-occipital alpha power are sensitive to unconscious semantic and response conflict changes (Jiang et al., 2015b). It has long been found that the neural oscillations of theta over medial frontal sites and alpha band over parieto-occipital sites are related to conflict and control processes (Nigbur et al., 2011; Cohen and van Gaal, 2013; Cavanagh and Frank, 2014; Cohen, 2014, 2017; Jiang et al., 2015a,b). Moreover, using a masked priming paradigm with non-affective primes and targets, fMRI studies revealed that the fronto-parietal networks including medial frontal cortex (MFC), dorsal lateral prefrontal cortex (DLPFC), and posterior parietal cortex (PPC) play a role in unconscious conflict detection and resolution (e.g., D’Ostilio and Garraux, 2012; Jiang et al., 2016). This neural network is similar to the one engaged by traditional cognitive control studies using consciously perceived stimuli in typical Stroop/Flanker tasks (e.g., MacLeod and MacDonald, 2000; Egner and Hirsch, 2005; van Veen and Carter, 2005).

In the current study, we aimed to examine the neural oscillatory dynamics of the unconscious conflict control mechanism in subliminal affective priming and the role of the frontal-parietal control network in resolving unconscious conflict. To realize our aim, we utilized a facial expression masked affective priming task combined with EEG time-frequency (TF) analysis methods. The use of facial expressions rather than other affective stimuli, such as affective words, was based on the following considerations: (1) compared to other affective stimuli, facial expressions are more natural and have a social and biological significance for survival (Andrews et al., 2011); and (2) perception and recognition of facial expressions are highly practiced and usually unconscious processes (Beall and Herbert, 2008). Therefore, larger priming conflict effects could be expected. TF spectral analyses rather than ERP measures were employed because most of the conflict-related EEG signal is non-phase-locked, and thus averaged out while calculating ERPs (Nigbur et al., 2011; Cohen and Donner, 2013). Moreover, the phase-based connectivity analyses in addition to the TF analyses of EEG would allow us to examine the interactions of different brain regions involved in conflict control. Based on previous studies, we hypothesized that the priming conflict would enhance the midfrontal theta and suppress parieto-occipital alpha activities (Jiang et al., 2015a,b), and that the fronto-parietal networks would play a great role in priming conflict control.

## Materials and Methods

### Participants

Twenty-two undergraduate students (17 females) between 18 and 24 (*M* = 21.95, *SD* = 1.43) years of age at the Third Military Medical University participated in this experiment for course credit or financial compensation. All participants were right-handed and had normal or corrected-to-normal vision. All experimental procedures were in line with the relevant laws and regulations and were approved by the ethical committee of the Third Military Medical University. In accordance with the approved guidelines, written informed consent was obtained from all participants after the explanation of the experimental protocol.

### Apparatus and Materials

Stimuli were presented against a black background at the center of a 21-inch Dell VGA monitor (frequency 70 Hz, resolution 1024 × 768) with the E-prime 2.0 software package (Psychology Software Tools, Pittsburgh, PA, USA).

Thirty-six sad and 36 happy facial expressions were selected from the new version of the Chinese Facial Affective Picture System (CFAPS; Gong et al., 2011), in which 12 sad and 12 happy pictures served as primes, and the others as targets. Each group of facial expressions were balanced between males and females. Moreover, Sad and happy facial expressions were matched to ensure they only differed significantly in scores of valences but not in arousal. For more information on the emotional rating of pictures please refer to our previous study (Jiang et al., 2013). The mask was a checkerboard pattern made in Photoshop. Participants viewed the stimulus from a distance of 70 cm. The entire prime or target subtended a visual angle of 8.82° × 9.26°.

### Experimental Procedures and Design

**Figure 1:**
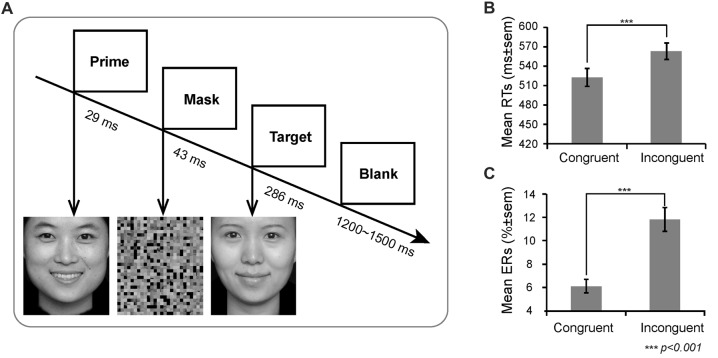
Experimental design and behavioral results. **(A)** Schematic representation of the task and stimuli. The valence of prime and target facial expressions could be congruent or incongruent (50/50 congruent/incongruent trials). The mean **(B)** Reaction times (RTs) and **(C)** Error rates (ERs) as a function of congruency.

On each trial, a prime face was first presented for 29 ms (2 frames), and then a mask appeared for 43 ms. Thereafter, a target face was presented for 286 ms (20 frames). Finally, a blank for a random duration ranging between 1200 ms and 1500 ms appeared (see Figure 1). To reduce the possibility that participants release their attention after each trial and wait for the fixation to reinstate their attentional focus (van Gaal et al., 2010), we did not include a fixation before the prime. Participants were instructed to respond as quickly and accurately as possible by pressing the key on the QWERTY keyboard to indicate the target valence. Half of the participants were instructed to press “F” with the left index finger if the target valence was positive and to press “J” with the right index finger if the target valence was negative; the finger-to-key mapping was reversed in the remainder of the participants. There were four types of prime-target pairs: happy-sad, happy-happy, sad-happy, sad-sad. These prime-target pairs were categorized as congruent (low conflict) and incongruent (high conflict) trials according to whether or not the valence between prime and target matched. All prime pictures were strongly masked (unconscious).

Participants completed 16 practice trials with feedback, followed by six test blocks of 96 trials each. In each block, the target faces were pseudo-randomly presented to ensure that each target only appeared twice in each block, once in each congruency condition, and none of the same target faces appeared in three consecutive trials. Participants were asked to take a break for 2 min with eyes closed after they performed each test block. After the test blocks, participants completed a two-alternative forced-choice task of 96 trials to discriminate the valence of the primes. In the discrimination task, the sequence, timing, and finger-to-key mapping of the stimuli were exactly the same as in the test blocks, except that the target was replaced by a response screen instructing the participants to indicate as accurately as possible whether a happy or a sad face was presented. Before completing the discrimination task, participants were told that the discrimination task had no time pressure, and that the frequency of happy and sad faces was equal.

### Behavioral Data Statistics

Incorrect trials and correct trials that fell outside three standard deviations (SD) of the mean RTs of each participant in each condition were excluded from all RT analyses (Jiang et al., 2013). Mean RTs and ERs were separately submitted to one-way repeated measures ANOVAs, with congruency (congruent vs. incongruent) as a within-subject variable. A one-sample *t*-test on d′, an objective bias-free measure of a subject’s ability to detect a signal, was used to analyze prime valence recognition. A two-tail significance level of 0.05 was used for all behavioral statistical tests.

### EEG Recordings

Participants sat in a dimly lit room and were instructed to avoid eye blinks, movements and muscle tension during stimulus presentation. EEG data were continuously recorded with a 64-channel BrainAmp amplifier (Brain Products, Munich, Germany) in an elastic cap based on the 10-20 system. During recording, all electrodes were referenced to FCz, and AFz served as the ground electrode. EEG signals were filtered using a 0.01–100 Hz band-pass and continuously sampled at 500 Hz. All electrode impedance was kept below 5 kΩ by careful preparation.

### EEG Preprocessing

All analyses were performed in MATLAB (R2015b, The MathWorks, Inc.) using custom made scripts supported by EEGLAB (Delorme and Makeig, 2004). Continuous EEG data were first offline re-referenced to the average of the activity recorded at the left and right earlobes and were digitally high-pass filtered at 0.5 Hz, and then segmented from −1.5 s to 2 s around target onset. The epochs corresponded to the behavioral exclusion criteria, and epochs deviating more than 5 SD from the mean probability distribution of potential values over all epochs and all channels were excluded. Next, independent component analysis (ICA) was performed to isolate artifacts in the EEG signal. Independent components representing eye blinks, muscle artifacts, or other types of noise were removed from the signal. After preprocessing, there were on average 263 congruent and 246 incongruent trials per condition per subject used in the following data analyses.

### Time-Frequency Decomposition

Before TF decomposition, all clean EEG data were transformed using a spatial filter called current-source-density (CSD) to minimize volume-conducted effects and to increase topographical selectivity by effectively removing spatially broad signals (Cohen and van Gaal, 2013). Single-trial EEG data for each condition were decomposed into their TF representations (TFRs) from 1 Hz to 40 Hz in 40 linearly spaced steps by first multiplying the power spectrum of the EEG (obtained from the fast Fourier transform) by the power spectrum of complex Morlet wavelets (e^*i*2π*tf*^ *e*^−*t*2^/^(2*σ*^2^)^), where *t* is time, *f* is frequency and *σ* defines the width of each frequency band, which was set as 3–7 logarithmically spaced cycles to trade-off temporal and frequency resolution, and then taking the inverse fast Fourier transform. Frequency band-specific power at each point in time was computed by taking the squared magnitude of the resulting complex signal (Cohen and van Gaal, 2013). In order to make the data comparable across all conditions, frequencies, and participants, power was normalized with a baseline from –350 ms to –150 ms before target onset, using a decibel (dB) transform. The baseline activity was taken as the average power at each frequency band, averaged across conditions (dB power = 10 × log 10[power/baseline]). In all analyses and plots, data are time-locked to target onset.

### Time-Frequency Power Analyses and Statistics

Our previous studies have revealed midfrontal theta and posterior alpha dynamics related to conflict processing (Jiang et al., 2015a,b), so we separately averaged across midfrontal (Fz, FC1, FCz, FC2, Cz), and occipito-parietal electrodes (P7, P5, P3, P1, Pz, P2, P4, P6, P8, Po7, Po3, Poz, Po4, Po8, O1, Oz, O2) as our regions of interest (ROIs), and then conducted *t*-tests. To control for multiple comparisons, cluster-based random permutation analyses were calculated to find clusters of significant activation at the spatial ROI (Maris and Oostenveld, 2007; Cohen and van Gaal, 2013), in which the assignment of condition to each data point was randomly shuffled, and statistics were re-computed. After thresholding each permutation map (*p* = 0.001), the number of pixels in the largest supra-threshold cluster was stored. This was repeated 2000 times, generating a distribution of maximum cluster sizes under the null hypothesis. Any clusters in the real data that were at least as large as 99% of the distribution of null hypothesis cluster sizes were considered statistically significant.

### RT–Power Correlation Analyses

Using Spearman rank correlations, we examined the association between the conflict effect (Incongruent-congruent) in RT and power averaged across trials. These correlations allowed us to test whether power was related to conflict as measured by RT across subjects. Then we examined the within-subject correlations between single trial RT and single trial power (trial-to-trial RT–power correlation) to test whether EEG TF dynamical features were related to trial-varying task performance as measured by RT over trials (Cohen and Donner, 2013; Cohen and van Gaal, 2014; Jiang et al., 2015b). This latter analysis produced a TF map of correlation coefficients for each subject and each condition. The correlation coefficients were tested against zero by *t*-tests at the group level after being Fisher-Z transformed because they are non-normally distributed. The results were corrected using the above mentioned cluster-based permutation method at *p* < 0.05 level.

### Connectivity Analyses

To examine whether fronto–partial networks were involved in unconscious conflict detection and resolution in a masked affective priming paradigm, we computed the debiased weighted phase lag index (dWPLI) using the *ft_connectivity_wpli* function in Fieldtrip toolbox (Oostenveld et al., 2011). The dWPLI is a measure of phase synchrony that is not affected by volume conduction and avoids the positive bias of WPLI (for more advantages and mathematical definition of this measure, please see Vinck et al., 2011; Phillips et al., 2014). Based on previous studies and the results of power analyses (Cohen and van Gaal, 2014), we selected the midfrontal FCz electrode as the “seed” to examine the inter-regional phase synchronization to other electrodes. The TF window was also based on the results of the power analyses.

### Granger Causality Analyses

TF Granger causality analysis (GCA) was used to further estimate the directional information flow of the temporal interactions among MFC, left DLPFC and PPC revealed by functional connectivity analyses mentioned above. The GCA was performed in the Fieldtrip toolbox (Oostenveld et al., 2011). Before this analysis, the EEG data were first down-sampled to 250 Hz to obtain a reasonable model order for multivariate autoregressive modeling (Seth, 2010), next the data were CSD transformed to reduce the impact of volume-conducted effects (Cohen and van Gaal, 2013; Wang et al., 2016), and finally the ensemble mean was removed from the data. Based on the functional connectivity analyses, we selected the MFC region channels (FCz), PPC region channels (Pz, P1, P3), and left DLPFC region channels (AF3, F3, F5) as our ROIs. The data in PPC and left DLPFC were averaged in time domain prior to GCA. For obtaining the time dimension of GCA results, we used slide windows over time for each trial and each condition. Due to the large number of time windows tested, it was difficult to select an optimal order based on Bayes information criterion or Akaike information criterion (Cohen and van Gaal, 2013). Therefore, we followed Cohen and van Gaal (2013) to select two sets of parameters (one with an order of 8 and a window of 200 ms and the other with an order of 12 and a window of 500 ms) in the GCA, and then averaged the results. The GCA results were normalized with a baseline of −350 to −150 ms before target onset. It is worth noting that we were not interested in the Granger causality influence *per se*, but rather the difference between conditions.

## Results

### Discrimination

Valence recognition of the masked primes as measured by *d*′ was not higher than zero (*M* = 0.002, *SD* = 0.09, *d*′ range: −0.18 to 0.15, *t*_21_ = 1.00, *p* = 0.91, corresponding to 50.09% correct on average). Consistent with our previous study (Jiang et al., 2013), participants were unable to recognize the valence of the primes consciously when they were strongly masked.

### Behavioral Emotional Priming Conflict

There was a main effect of prime-target congruency for both response time and ERs (RT: *F*_(1,21)_ = 140.47, *p* < 0.001; ER: *F*_(1,21)_ = 63.61, *p* < 0.001), revealing that participants responded far slower and made more errors on incongruent trials (RT: *M* = 563 ms, *SE* = 12.41; ER: *M* = 11.82%, *SE* = 1.00%) than on congruent trials (RT: *M* = 523 ms, *SE* = 13.93; ER: *M* = 6.12%, *SE* = 0.57%, Figure 1B).

### Time–Frequency EEG Power

**Figure 2:**
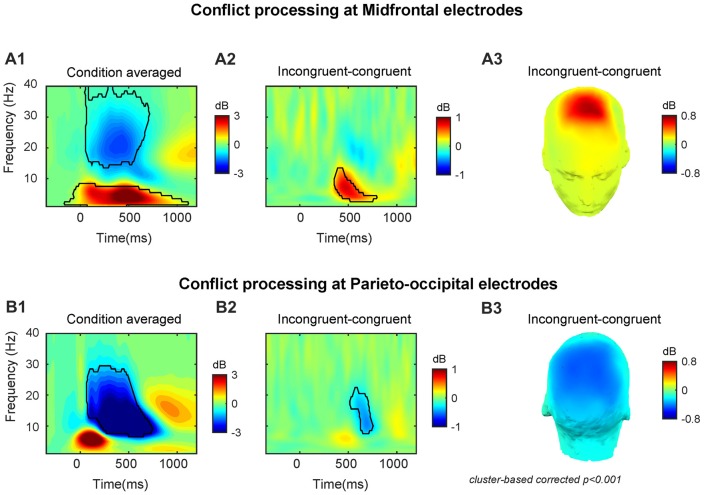
Conflict processing reflected in neural oscillations at midfrontal and parieto-occipital electrodes. The time-frequency (TF) power dynamics plots for **(A1)** condition averaged and **(A2)** the conflict effect, and **(A3)** topographic plots of the conflict effect at midfrontal electrodes. The TF power dynamics plots for **(B1)** condition averaged and **(B2)** the conflict effect neural oscillations and **(B3)** topographic plots of the conflict effect at parieto-occipital electrodes. Time 0 is the onset of target facial expression. The TF region encircled by the black solid line is statistically significant at *p* < 0.001 (corrected for multiple comparisons using cluster based statistics). Panels **(A3,B3)** were plotted using the averaged data enclosed area in panels **(A2,B2)**.

At the midfrontal region, the target-onset-locked TF power dynamics revealed increased theta/delta activity and decreased beta activity in both congruent and incongruent conditions (Supplementary Figures S1A1,2). At the parietal-occipital region, TF power dynamics revealed a strongly increased theta activity and a suppressed alpha/beta activity for both congruent and incongruent conditions (Supplementary Figures S1B1,2). The across conditions averaged midfrontal activity (peak power = 3.87 dB, peak frequency = 4 Hz, peak time = 490 ms, frequency range: 1–8 Hz, time range: −160–1110 ms, Figure 2A1) and parieto-occipital activity (peak power = −6.39 dB, peak frequency = 11 Hz, peak time = 520 ms, frequency range: 7–29 Hz, time range: 70–740 ms, Figure 2B1).

In line with our previous studies (Jiang et al., 2015a,b), we observed that the conflict effect (incongruent-congruent) resulted in enhanced midfrontal theta power (peak power = 0.85 dB, peak frequency = 7 Hz, peak time = 470 ms, frequency range: 3–13 Hz, time range: 360–780 ms, Figures 2A2,3) and decreased parieto-occipital alpha power (peak power = −0.50 dB, peak frequency = 11 Hz, peak time = 690 ms, frequency range: 8–22 Hz, time range: 520–750 ms, Figures 2B2,3).

### RT–Power Correlations

**Figure 3:**
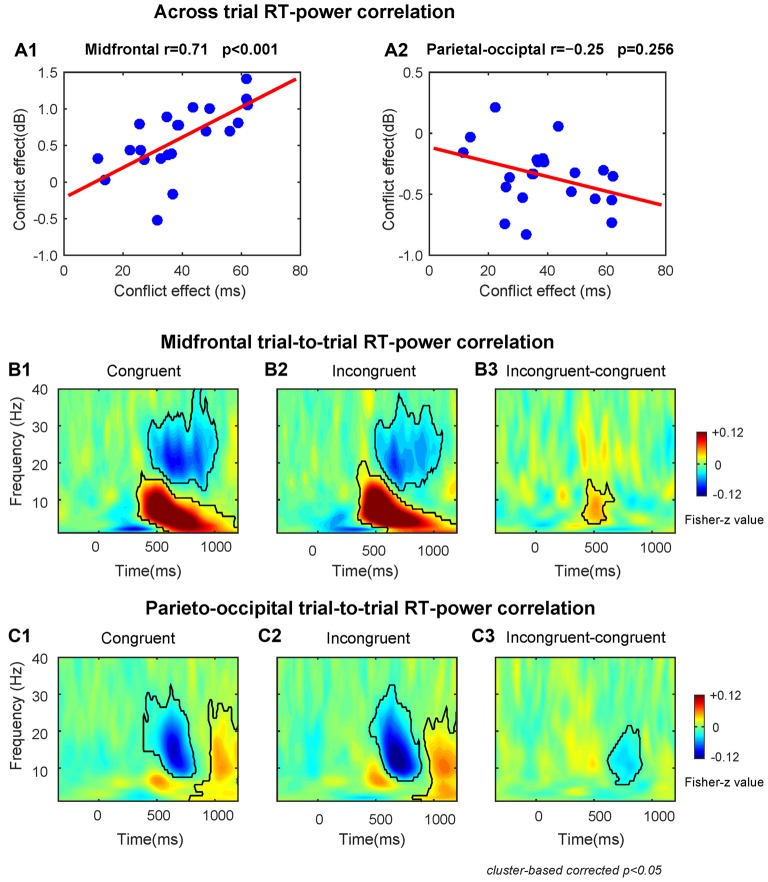
The results of RT-power correlations. **(A1)** The midfrontal theta power was significantly positively correlated with the conflict effect on RT. **(A2)** The correlation between parieto-occipital alpha power and the conflict effect on RT was not significant. The TF plots for the correlation between single-trial RT and single-trial power on congruent **(B1)** and incongruent **(B2)** conditions and the conflict effect on correlation coefficient **(B3)** at midfrontal region of interest (ROI) electrodes. The TF plots for the correlation between single-trial RT and single-trial power on congruent **(C1)** and incongruent **(C2)** conditions and the conflict effect on correlation coefficients **(C3)** at parieto-occipital ROI electrodes. Each pixel in TF plots (panels **B1–3,C1–3**) represent the Fisher-Z transformed Spearman correlation coefficient. Black contours in TF plots outline regions that are statistically significant at *p* < 0.05 and corrected for multiple comparisons using cluster based statistical methods.

The results of the across-subject RT-power correlation analysis revealed that the conflict effect on theta power was significantly (*r* = 0.71, *p* < 0.001) and positively correlated with the conflict effect on RT at the midfrontal region (Figure 3A1). The conflict effect on alpha power was not significantly (*r* = −0.25, *p* = 0.256) correlated with the conflict effect on RT at the parieto-occipital region (Figure 3A2).

The within-subject trial-to-trial RT-power correlation results are shown in Figures 3B,C. We found that at the midfrontal region during congruent and incongruent trials there was a significant positive correlation in the delta/theta band (Congruent: peak frequency = 6 Hz, time range: 330–1200 ms; Incongruent: peak frequency = 7 Hz, time range: 340–1200 ms), and a significant negative correlation in the beta band (Congruent: peak frequency = 20 Hz, time range: 430–1020 ms; Incongruent: peak frequency = 20 Hz, time range: 500–1070 ms, Figures 3B1,2). At the parieto-occipital region, there was a significant negative correlation in the alpha/beta band (Congruent: peak frequency = 15 Hz, time range: 390–830 ms; Incongruent: peak frequency = 13 Hz, time range: 450–890 ms) and a significant positive correlation in the alpha band (Congruent: peak frequency = 10 Hz, time range: 780–1200 ms; Incongruent: peak frequency = 10 Hz, time range: 820–1200 ms, Figures 3C1,2).

Moreover, the correlation coefficients of incongruent trials were larger than congruent trials in midfrontal theta band (peak frequency = 8 Hz, time range: 400–670 ms, Figure 3B3) and smaller in parietal-occipital alpha band (peak frequency = 11 Hz, time range: 620–910 ms, Figure 3C3). Therefore, the fluctuations of conflict on RTs across trials is also associated with the midfrontal theta and parieto-occipital alpha band power related to conflict, replicating the results of the across-subject correlations described above.

### Fronto–Parietal Synchronization-Based Conflict Control Networks

**Figure 4:**
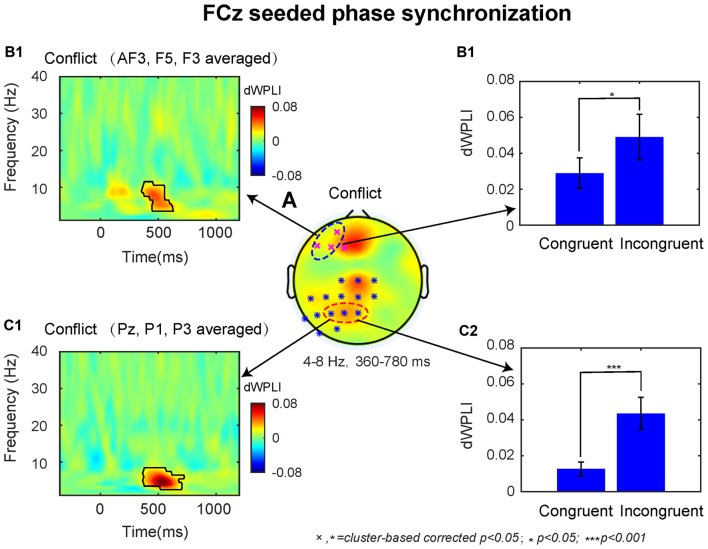
The results of connectivity analyses. **(A)** The conflict effect at the FCz seeded phase synchronization in the time-window of 360–780 ms revealed two statistical clusters at *p* < 0.05. The TF plot **(B1)** and bar plot **(B2)** as a function of congruency across left dorsal lateral prefrontal cortex (DLPFC) electrodes (AF3, F5, F3). The TF plot **(C1)** and bar plot **(C2)** as a function of congruency across left posterior parietal cortex (PPC) electrodes (Pz, P1, P3). Each pixel in TF plots represent the debiased weighted phase lag index (dWPLI) values. Black contours in TF plots outline regions that are statistically significant at *p* < 0.05 and corrected for multiple comparisons using cluster based statistics methods.

The dWPLI TF window (4–8 Hz, 360–780 ms) was selected based on the conflict effect on power over the MFC region (Figures 2A2,3). The cluster-based permutation tests on the averaged data revealed a significant difference between the incongruent and congruent condition (*p* < 0.05), which was pronounced over left lateral prefrontal and parietal electrodes (Figure 4A).

Based on previous studies, we selected the significant electrodes located at left DLPFC (AF3, F3, F5) and PPC (Pz, P1, P3) in the fronto-parietal network for subsequent analyses. The TF plots for the dWPLI data shown in Figures 4B1,C1 revealed that the phase synchrony from MFC to left DLPFC and PPC sites was mainly confined to theta band. Within the TF window (4–8 Hz, 360–780 ms) from the electrodes selected above, the data revealed stronger theta band phase synchronization for incongruent than congruent trials (left DLPFC: *M* = 0.02, *t* = 2.59 *p* = 0.017, Figure 4B2; PPC: *M* = 0.03, *t* = 4.20, *p* < 0.001; Figure 4C2).

### Granger Results

**Figure 5:**
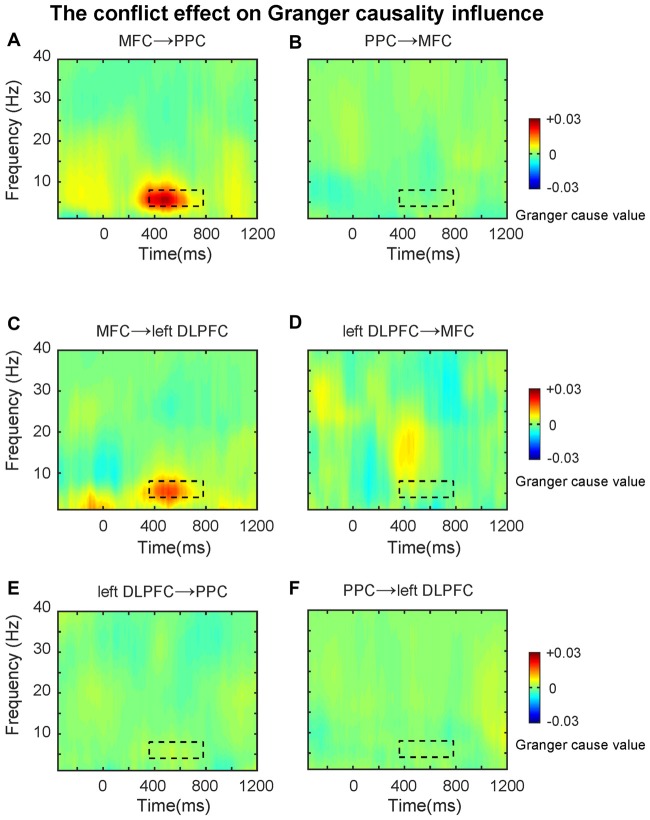
The conflict effect on the Granger causality influence. TF plots for the conflict effect for **(A)** MFC→PPC; **(B)** PPC→MFC; **(C)** MFC→left DLPFC; **(D)** left DLPFC→MFC; **(E)** left DLPFC→PPC; **(F)** PPC→left DLPFC directed synchrony. The black dotted line denotes the time frequency window (4–8 Hz, 360–780 ms, based on the results from connectivity analysis), and data within this area were averaged and extracted to perform the ANOVAs. The Granger causality analysis primarily revealed the top-down directed influence involved in conflict control during subliminal affective priming.

First, we tested directional synchronization between MFC and PPC sites. A repeated ANOVA was performed on the data within the TF window (4–8 Hz, 360–780 ms, based on the results from connectivity analysis) with direction (MFC→PPC vs. PPC→MFC, Figures 5A,B) and congruency (Congruent vs. Incongruent) as within-subjects factors. The ANOVA results showed that theta-band directed synchrony in incongruent trials was stronger than in congruent trials (*F*_(1,21)_ = 10.40, *p* = 0.004), and theta-band directed synchrony in MFC→PPC was significantly stronger than in PPC→MFC (*F*_(1,21)_ = 11.23, *p* = 0.003). The interaction between direction and congruency was significant (*F*_(1,21)_ = 9.01, *p* = 0.007). Further analyses showed that the conflict effect (Incongruent-congruent, *M* = 0.01, *t* = 3.16, *p* = 0.005) on MFC→PPC theta-band directed synchrony was statistically significant, while conflict on PPC→MFC theta-band directed synchrony was not significant (*M* = 0.00, *t* = 0.50, *p* = 0.619). These finding are consistent with the idea that MFC region uses theta oscillation to send top-down control signals (Cohen and van Gaal, 2013).

Because previous studies also showed that the PFC has a greater role in top-down conflict control (Cohen and van Gaal, 2013), in the following analysis we tested the theta-band directed synchrony between left DLPFC and MFC with the same factors and TF windows mentioned above. The results revealed that the directed synchrony in incongruent trials was stronger than in congruent trials (*F*_(1,21)_ = 5.20, *p* = 0.033), while there was no significant difference between MFC→left DLPFC and left DLPFC→MFC (Figures 5C,D). The interaction was marginally significant (*F*_(1,21)_ = 3.52, *p* = 0.074). Follow-up analyses revealed that the conflict effect (*M* = 0.01, *t* = 2.21, *p* = 0.038) on MFC→left DLPFC theta-band directed synchrony was significant, while the conflict effect on left DLPFC→MFC theta-band directed synchrony was not significant (*M* = 0.00, *t* = 0.32, *p* = 0.75).

In the next analyses, we tested the theta-band directed synchrony between left DLPFC and PPC. As shown in Figures 5E,F, the conflict effect was not significant in left DLPFC→PPC or PPC→left DLPFC. The data was tested within the TF window (4–8 Hz, 360–780 ms, based on the results from connectivity analysis) with direction (left DLPFC→PPC vs. PPC→left DLPFC) and congruency (Congruent vs. Incongruent) as within-subject factors, revealing no significant effects (*p*’s > 0.05).

## Discussion

The current study utilized a facial expression version of the masked affective priming task combined with EEG TF data analysis to investigate the neural oscillatory dynamics of conflict control during subliminal affective priming. The behavioral data revealed a typical affective congruency effect; participants responded slower and were more error-prone on incongruent trials than congruent trials, even though the primes were unconsciously presented, replicating our previous study. Specifically, using a subliminal facial affective priming task, in which a single masked happy/sad facial expression prime precedes a supraliminal happy/sad facial expression target, we observed that the RTs and ERs for judgments of the targets’ valence were higher on incongruent trials relative to congruent trials despite participants lack of conscious awareness of the primes’ valence (Jiang et al., 2013). Due to the similarity between the affective congruency effect and typical cognitive conflict effects in tasks such as the Stroop or Flanker, previous studies often described affective congruency effect as being analogous to classic cognitive conflict (Klauer and Musch, 2003; Frings and Wentura, 2008). That is, the affective congruency effect may originate from interference between the semantic meaning/valence of the primes and targets at the stimulus level and/or the response competition level. While previous studies have offered this explanation, the exact source of conflict in the affective priming paradigms was still debated (Klauer and Musch, 2003; Bartholow et al., 2009; Bartholow, 2010).

The TF power dynamics analyses revealed that the conflict evoked a midfrontal theta power increase and a parieto-occipital alpha power decrease. Moreover, the behavioral-brain correlations illustrated that the conflict-related midfrontal power varied as a function of conflict in RT. The results of the single trial correlation further showed that longer RTs were associated with increased midfrontal power in theta and decreased parieto-occipital power in alpha, and that the correlation coefficient was larger in incongruent trials than congruent trials over both regions. Overall, the results of the power dynamics analyses, suggest that midfrontal theta and parieto-occipital alpha are related to conflict processing in subliminal affective priming.

Our findings fits well with previous EEG studies using typical conflict tasks and masked priming tasks (Jiang et al., 2015a,b). Prior work has reported that the neural response to conflict is an increase of MFC theta band activity (Jiang et al., 2015a,b). In particular, research has demonstrated that conflict results in enhances MFC theta activity, which serves as a signature of semantic conflict (Jiang et al., 2015b), or conflict at the stimulus and response levels (Nigbur et al., 2012). It has also been associated with error related processing after conflicts (e.g., Cavanagh et al., 2009). The current study’s finding are also in line with work using ERP measures to reveal affective priming conflict increases conflict-related fronto-central N2 amplitude (Bartholow et al., 2009; Eder et al., 2012). The conflict-related N2 component is partially a reflection of the theta oscillatory process (e.g., Cavanagh et al., 2012). The MFC theta oscillations have often been regarded as a marker of conflict processing, and may be a reflection of a general conflict detection and the monitoring mechanism of the MFC (Cavanagh et al., 2009; Nigbur et al., 2011, 2012; Cohen and Donner, 2013; Cohen and van Gaal, 2013; Cavanagh and Frank, 2014; Cohen, 2014; Jiang et al., 2015b). In short, the functional implication of MFC theta in cognitive control may be to provide a reference system for conflict monitoring and adjusting subsequent behaviors (Cohen, 2014).

Consistent with the current study, our previous work using masked priming paradigms have also found a decrease in alpha power over parieto-occipital electrodes on incongruent trials compared to congruent trials (Jiang et al., 2015a,b). Moreover, the alpha power decrease at posterior parietal regions after conflict is also consistent with previous studies on the functional role of alpha band oscillations in conflicts/errors (Carp and Compton, 2009; Compton et al., 2011; van Driel et al., 2012; Cohen and Ridderinkhof, 2013; Jiang et al., 2015a,b). It has been suggested that the reduction of alpha activities over posterior parietal regions may reflect either a more general attention mechanism (van Driel et al., 2012; Cohen and Ridderinkhof, 2013) or inhibitory control of prepotent motor response tendencies (Hwang et al., 2014; Sadaghiani and Kleinschmidt, 2016) during cognitive control. These interpretations of the functional role of alpha oscillations fits well with our finding that alpha power showed more suppression around 520–750 ms on incongruent than congruent trials (Figures 2B2,3). To resolve conflict, participants may increase motor control by increasing alertness on the next trial (Carp and Compton, 2009; Compton et al., 2011; van Driel et al., 2012). Specifically, to inhibit pre-activated motor response tendencies, the task-irrelevant stimulus should be inhibited and/or alertness and refocusing of attention to task-relevant stimulus in the next trial should be increased (van Driel et al., 2012; Cohen and van Gaal, 2013; Jiang et al., 2015b). Further, the motor control and response selection during conflict resolution has long been linked to the function of PPC (Cohen and Ridderinkhof, 2013). Previous fMRI studies have suggested that PPC regions are involved in the resolution of conflict in various forms, including Stroop-like conflict (e.g., Jiang et al., 2016) and Flanker-like conflict (e.g., D’Ostilio and Garraux, 2012) in masked priming tasks.

The results of MFC-centered functional connectivity analyses showed that phase synchronization between MFC and left DLPFC and between MFC and PPC were enhanced after conflict, and this functional connectivity in the fronto-parietal network was restricted to theta band oscillations. These findings suggested that the fronto-parietal network is involved in conflict control. During conflict control processes, theta band oscillations over MFC may reflect a common neural computation mechanism to be used as a physiological signal to communicate between brain regions and signal the need for conflict control (Cavanagh et al., 2009; Nigbur et al., 2011, 2012; Cohen and Donner, 2013; Cohen and van Gaal, 2013; Cavanagh and Frank, 2014; Cohen, 2014). According to the conflict monitoring model, the MFC detects conflict and propagates the signals to the DLPFC to execute cognitive control (Botvinick et al., 2001). Further, Walsh et al. (2011) have demonstrated that the conflict processing in MFC regions interacts with attentional control in PPC regions to implement post-conflict behavioral adjustments. Therefore, the brain regions in the fronto-parietal network may work in concert to implement cognitive control using theta band phase synchronization.

The results of the GCA extended the MFC-centered theta phase synchronization by demonstrating that the conflict-driven information flow in the fronto-parietal conflict control network was unidirectional. The data revealed that only MFC→PPC and MFC→left DLPFC were statistically significant. The GCA as a confirmatory analysis further demonstrated that the theta band phase synchronization plays a prominent role in top-down conflict control. The findings of the GCA fits well with the conflict monitoring model (Botvinick et al., 2001), because the MFC→left DLPFC directional synchronization may reflect that the MFC detects conflicts and signals to the DLPFC to exercise top-down conflict control. The MFC→PPC directional synchronization suggests that the MFC may also directly implement some aspects of behavioral adjustment by modifying the motor response and sensory processing in the PPC regions (Cohen and van Gaal, 2013). That is, to control conflict the MFC might implement top-down conflict control by signaling the PPC to bias sensory processing towards the goal-relevant information and inhibit the incorrect/prepotent motor response.

In conclusion, the neural correlates of conflict processing in subliminal affective priming involve the midfrontal theta band and posterior parietal alpha band oscillations. The theta band phase synchronization in the frontal-parietal network is involved in top-down conflict resolution in the subliminal affective prime conflict. Therefore, the mechanism of the subliminal affective priming effect is similar to classic cognitive conflict, and our findings provide a new perspective to explain the nature of the subliminal affective priming effect.

## Author Contributions

JJ designed the experiment and analyzed the data. JJ, KB, and XX wrote the article.

## Conflict of Interest Statement

The authors declare that the research was conducted in the absence of any commercial or financial relationships that could be construed as a potential conflict of interest.

## Supplementary Material

FIGURE S1 Task-related time–frequency (TF) power plotted separately for each condition. The TF plots of power dynamics for **(A1)** congruent and **(A2)** incongruent condition at midfrontal electrodes. The TF plots of power dynamics for **(B1)** congruent and **(B2)** incongruent condition at parieto-occipital electrodes.
