# Supplementary figures and images for: Midfrontal Theta and Posterior Parietal Alpha Band Oscillations Support Conflict Resolution in a Masked Affective Priming Task
Source: Front Hum Neurosci. 2018 May 3;12:175. doi: 10.3389/fnhum.2018.00175 (PMC5943601; doi:10.3389/fnhum.2018.00175)

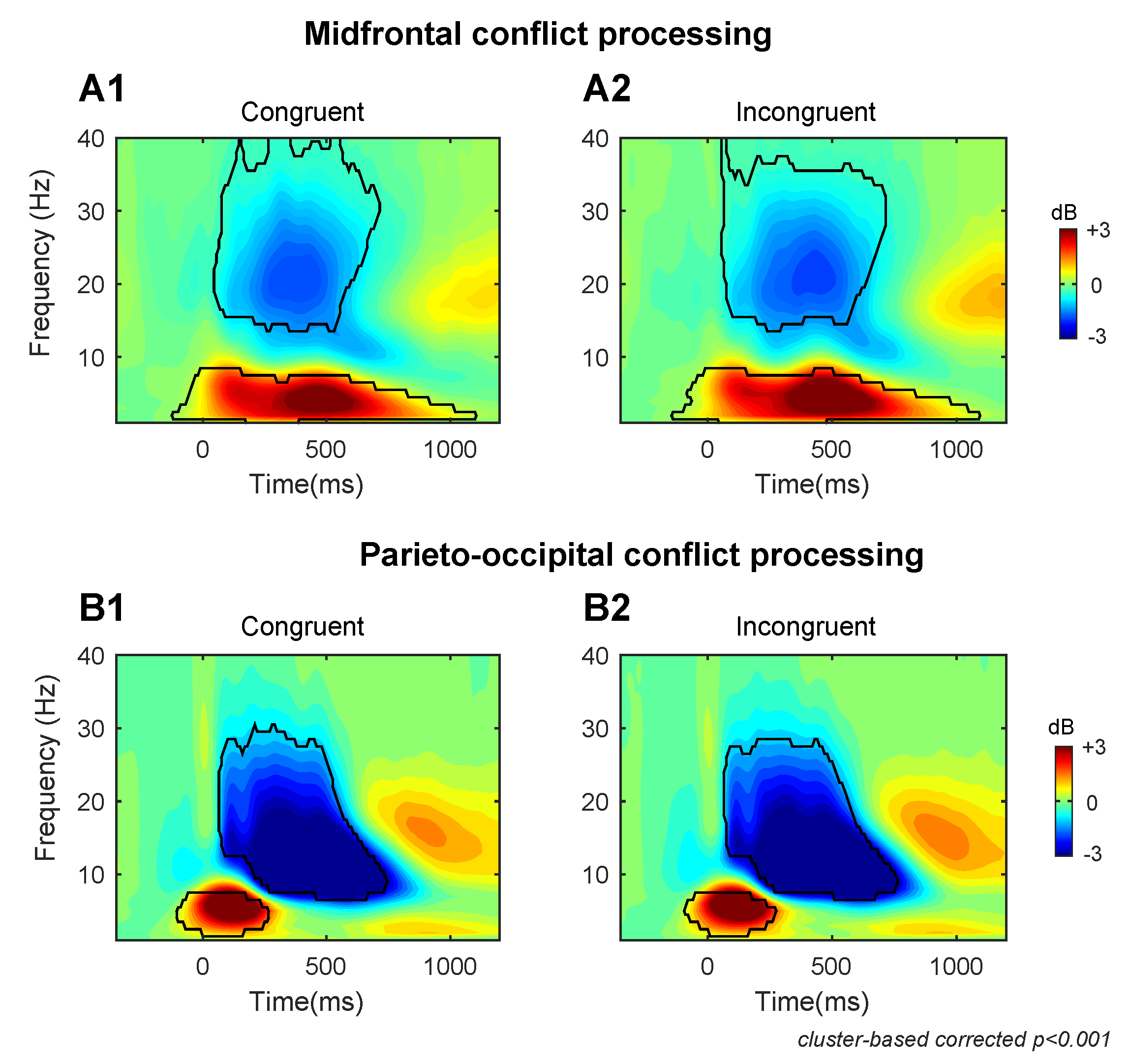

Supplement: FIGURE S1 — Task-related time–frequency (TF) power plotted separately for each condition. The TF plots of power dynamics for (A1) congruent and (A2) incongruent condition at midfrontal electrodes. The TF plots of power dynamics for (B1) congruent and (B2) incongruent condition at parieto-occipital electrodes. [file Image_1.TIF]
